# Alcohol and Metabolic Stress Synergize to Dysregulate Mitochondrial Health and Lipid Metabolism; Evidence from a Hepatocyte Spheroid Model

**DOI:** 10.1093/function/zqaf049

**Published:** 2025-11-03

**Authors:** Eden M Gallegos, Kaitlin Couvillion, Drake Darden, Keishla Rodriguez-Graciani, Patricia E Molina, Liz Simon

**Affiliations:** Department of Physiology, Louisiana State University Health Sciences Center, New Orleans, LA, 70112, USA; Department of Physiology, Louisiana State University Health Sciences Center, New Orleans, LA, 70112, USA; Department of Physiology, Louisiana State University Health Sciences Center, New Orleans, LA, 70112, USA; Department of Physiology, Louisiana State University Health Sciences Center, New Orleans, LA, 70112, USA; Department of Physiology, Louisiana State University Health Sciences Center, New Orleans, LA, 70112, USA; Department of Physiology, Louisiana State University Health Sciences Center, New Orleans, LA, 70112, USA

**Keywords:** hepatocyte, alcohol, metabolism, steatosis, MetALD, mitochondria

## Abstract

Metabolic dysfunction-associated steatotic liver disease and alcohol-associated liver disease frequently co-occur, manifesting as MetALD. Understanding the hepatocyte-specific effects of alcohol and metabolic stressors is critical to uncovering mechanisms of synergistic injury. This study evaluated the individual and combined effects of ethanol, sugars, and saturated/monounsaturated fats on hepatocyte lipid metabolism, oxidative stress, and mitochondrial function using a 3D human HepaRG spheroid model. HepaRG spheroids were treated with ethanol (50 mM), sugar (glucose and fructose), and fatty acids alone or in combination for 10 d. The combination of ethanol (E) and metabolic (sugar and fat, SF) stressors (ESF) synergistically increased triglyceride content and lipid droplet accumulation. ESF increased gene expression of lipid handling targets including perilipins 1 and 2, fatty acid binding protein 1, and hepatic lipase compared to controls. ESF also induced the highest rate of ROS production compared to E and SF and dysregulated antioxidant gene expression. E and SF additively impaired ATP content and ATP production linked mitochondrial respiration. Ethanol and metabolic stressors synergize to dysregulate hepatocyte lipid homeostasis and oxidative stress while additively impairing mitochondrial bioenergetics. Gene expression results suggest that lipid accumulation may be driven by altered expression of triglyceride storage and lipid handling markers rather than de novo lipogenesis. These findings highlight the importance of metabolic contributions in alcohol-induced hepatocellular dysfunction and establish HepaRG spheroids as a robust model to elucidate hepatocyte-specific responses in MetALD.

## Graphical Abstract

**Graphical Abstract:**
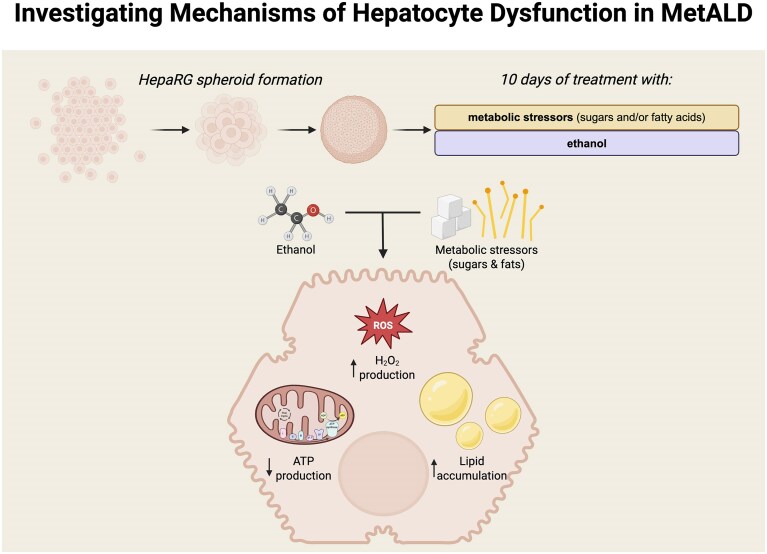

## Introduction

Steatotic liver diseases (SLDs) are a spectrum of pathologies—including hepatic lipid accumulation, inflammation, and fibrosis—that are increasingly prevalent worldwide.^1-3^ SLDs are further classified as metabolic-associated SLD (MASLD), alcohol-associated SLD (ALD), and their combination, MetALD.^4^ Alcohol-associated SLDs have higher morbidity and mortality risks than other SLDs, while MASLD is more prevalent than ALD.^5-8^ However, it is challenging to identify the primary driver of injury in MetALD.^9^ Thus, a recent focus in the field is understanding the compounding or unique pathophysiology in pre-clinical models and using cell culture models of major cell types implicated in MetALD pathogenesis.^10^

Our group and others have shown alcohol-induced alterations to both lipid and glucose metabolism in the liver of rodents and non-human primates, as well as mitochondrial bioenergetic impairments.^11-14^ Additionally, alcohol-associated alterations to metabolic pathways may be accentuated in the presence of compounding stressors such as inflammation or metabolic stressors.^15,16^ The liver simultaneously coordinates detoxification, nutrient sensing, and energy homeostasis, placing it in a central but vulnerable position with chronic alcohol exposure. Hepatocytes specifically are the primary site of ethanol metabolism, making them uniquely vulnerable to alcohol-induced injury.^17^ The pathways of alcohol metabolism—including via alcohol and acetaldehyde dehydrogenases, CYP2E1, and catalase—place an immense cellular burden on hepatocytes and trigger metabolic reprogramming that impacts lipid storage, mitochondrial bioenergetics, and antioxidant defenses.^17-20^ In animal studies which aimed to model MetALD, massive immune cell infiltration was a large contributor to steatosis induction, and thus, the direct effects of alcohol and high-fat/sugar diets on the hepatocytes may be difficult to elucidate.^21-25^ Hepatocyte injury and bioenergetic dysregulation are key drivers of ALD progression. However, nonparenchymal or infiltrating cell types can heavily influence the bioenergetic function of hepatocytes. Therefore, in this study we aimed to establish hepatocyte-specific direct metabolic alterations from ethanol and metabolic stressors.

While alcohol alone is sufficient to induce hepatocellular stress, its impact is highly dependent on nutritional confounders.^26^ Fructose and ethanol both upregulate de novo lipogenesis (DNL) through overlapping transcriptional pathways, including sterol regulatory element-binding protein 1 (SREBP1) and carbohydrate-responsive element binding protein (CHREBP), while saturated fatty acids interfere with mitochondrial fatty acid oxidation.^27-30^ Ethanol and dietary substrates compete for NAD+, produce acetyl-CoA, and challenge mitochondrial oxidative capacity, creating a metabolic competition for electron-accepting cofactors that promote steatosis and impair adaptive stress responses.^17,31^ Recent studies suggest that the combination of dietary excess and alcohol exposure can amplify lipid accumulation and redox dysfunction through synergistic disruption of lipid homeostasis.^22,25^,^32-34^

Mitochondrial dysfunction and oxidative stress are central mechanisms in both ALD and MASLD pathogenesis.^33^,^35-37^ Ethanol metabolism generates reactive oxygen species (ROS) via CYP2E1 activity and impairs antioxidant defenses, while chronic nutrient overload induces similar redox stress through lipid metabolite accumulation and insulin resistance.^18,38,39^ These processes converge in the mitochondria, which play a critical role in hepatic ATP synthesis, fatty acid oxidation, and redox regulation.^35,39^ Human studies of ALD and MASLD reveal reduced hepatic ATP levels and mitochondrial DNA damage, but how mitochondria respond to the combined burden of ethanol and metabolic substrates remains unknown.^33,35,36,40,41^

To study these interactions at the hepatocyte level, we sought a physiologically relevant in vitro model capable of sustaining both ethanol metabolism and nutrient responsiveness.^42^ Some hepatoma lines, such as HepG2, lack stable ADH and CYP2E1 expression, while primary hepatocytes rapidly dedifferentiate and lose function over time.^43-45^ HepaRG cells, on the other hand, retain stable expression of ethanol-metabolizing enzymes and demonstrate resistance to excess lipid accumulation when challenged with fatty acids alone.^46-48^ Cultured as 3D spheroids, HepaRG cells exhibit improved albumin secretion and enzyme expression compared to monolayer cultures and are appropriate for mitochondrial bioenergetic studies.^46^ Further, primary hepatocyte 3D spheroids show greater global proteomic similarity to donor human liver tissue as compared to monolayer cultures from the same donor.^44^ Thus, this model provides a robust platform to mechanistically dissect the additive and synergistic effects of ethanol and metabolic substrates on hepatocyte physiology.

The goal of this study was to evaluate the individual and combined effects of ethanol, sugars, and fats with a focus on hepatocyte lipid metabolism, oxidative stress, and mitochondrial function using a 3D HepaRG human spheroid model. We hypothesized that metabolic stressors and ethanol would have additive effects on lipid accumulation, and that their combination would synergistically impair mitochondrial function and oxidative stress regulation.

## Methods

### Chemicals and Media Preparation

#### Fatty Acid Conjugation

Fatty acids were prepared for cell culture as published^49^ with the following modifications. Sodium oleate (O7501-1 G, Sigma) and sodium palmitate (P9767-5 G, Sigma) were conjugated to fatty acid-free bovine serum albumin (BSA; A3803, Sigma) at a 5.28 fatty acid to 1 BSA ratio by constant stirring overnight at 37°C and sonication of the palmitic acid preparation. Vehicle BSA preparations were prepared and treated similarly for both the oleic acid and palmitic acid conjugate preparations. The final stock preparations were 8 mM oleic acid in 10% BSA and 4 mM palmitic acid in 5% BSA.

#### Plating Media

Plating media was prepared using William’s E Medium (US Biological Life Sciences, W1105-02) with 2 mM glutaMAX (ThermoFisher, 35050061), 10% heat-inactivated FBS, 5µg/mL insulin, 50µm Hydrocortisone 21-hemisuccinate sodium salt (HHS; H2270, Sigma), 100 Units/mL penicillin, 100µg/mL streptomycin, 1.7% DMSO, 5.5 mM glucose (G8644, Sigma), and 0.227 mM sodium pyruvate (Agilent 103578-100).^50-52^ The plating medium pH was adjusted to 7.4 and sterile filtered using a 0.22 μm vacuum filter before use.

#### Experimental Media

Control maintenance media contained William’s E Medium (US Biological Life Sciences, W1105-02) with 1X Maintenance and Metabolism media supplement (HPRG720, ThermoFisher), 2 mM glutaMAX (ThermoFisher, 35050061), 0.227 mM sodium pyruvate, 0.75% fatty acid-free BSA (vehicle), and 5.5 mM glucose (G8644, Sigma). High sugar media was prepared with 11 mM glucose and 11 mM fructose (F3510, Sigma). High-fat media was prepared with 300 μm of both oleic acid and palmitic acid (total BSA: 0.75%). Ethanol media was adjusted to a final concentration of 50 mM ethanol. Media were brought to a pH of 7.4 and sterile filtered using a 0.22 μm vacuum filter before use. Treatment groups included: control (C), ethanol (E), sugar-fat (SF), ethanol sugar-fat (ESF), sugar (S), ethanol sugar (ES), fat (F), and ethanol fat (EF).

### Cell Culture

Terminally differentiated HepaRG cells (HPRGC10, ThermoFisher) were thawed and plated according to the manufacturer’s protocols. Briefly, 10 × 10^6^ cells were thawed and assessed for cell viability using the Trypan blue exclusion method. To facilitate spheroid formation, cells in plating media were seeded at 2000 cells per well in ultra-low attachment plates (174925, ThermoFisher) and centrifuged at 200 × *g* for 1 min before incubation at 37°C and 5% CO_2_. Spheroids were allowed to form for 3 to 5 d in plating media and then switched to maintenance or experimental media. Thereafter, spent media was replaced every 3-4 d for a total of 10 d. At endpoint, spheroids were used for assays or processed and collected for downstream analysis. Supernatant (50 μL) was collected in duplicate from 10 wells and pooled at study endpoint.

### Spheroid Size Quantification

Brightfield images were used to measure spheroid diameter using the ImageJ program with a global image scale of 0.6097 pixels/μm. Three different diameter vertices were measured for each spheroid and averaged.

### DNA Isolation

DNA was isolated using the DNeasy Blood & Tissue kit (69504, Qiagen) according to the manufacturer’s instructions and quantified using the AccuClear Ultra High Sensitivity dsDNA Quantification Kit (31028, Biotium).

### Protein Expression

Protein expression determination was completed using methods described previously with adaptations.^11^ Five spheroids per experimental group were washed in 1X PBS pH 7.4 and stored at −80°C for protein extraction. Spheroids were lysed in 30 μL of 1X RIPA buffer with HALT protease and phosphatase inhibitor and 5 mM EDTA and briefly homogenized using a motorized pestle. Samples were centrifuged (10 000 × *g*, 10 min, 4°C) and 20 μL of supernatant was combined with lysis buffer, Laemmli loading buffer, and beta-mercaptoethanol at a final volume of 40 μL. Twenty microliter was heated at 95°C for 5 min, and the remaining 20 μL was heated at 37°C for 30 min. Samples were placed on ice, vortexed, centrifuged, and run on 4-15% polyacrylamide gels (BioRad, 4561083) at 120 V for 1 h. Gels were transferred to activated polyvinylidene difluoride (PVDF) membranes (Millipore, IPFL00010) in transfer buffer (dH2O/Tris/Glycine/methanol) overnight at 30 V and 4°C. Non-specific proteins were blocked using 5% BSA in TBS-T on a rocker for 1 h at RT. Protein membranes were incubated in a primary antibody solution at a concentration of 1:1000 in 3% BSA TBS-T overnight at 4°C. Protein membranes were incubated on a rocker for 1 h RT with anti-rabbit or anti-mouse conjugate HRP secondary antibodies, as appropriate, at a concentration of 1:10 000 in 3% BSA TBS-T. Protein bands were visualized with enhanced chemiluminescence (Millipore, WBLUF0500) for 5 min using an Asherman Imager 680.

### Albumin Secretion Quantification

Albumin secretion was measured using the Human Albumin ELISA kit (EHALB, ThermoFisher). The last media change occurred 3 d before supernatant collection. Briefly, at endpoint, supernatant was collected from 10 wells (50 μL per well), pooled, and stored at −80°C. The supernatant was diluted 10-fold prior to use with the ELISA kit according to the manufacturer’s directions. Albumin levels were quantified using a standard curve, normalized to 2000 cells, and divided by 3 to calculate daily albumin secretion rate per cell.

### Gene Expression

Spheroids were homogenized using the Qiazol homogenization and extraction method, and RNA was purified using the Qiagen miRNeasy micro-RNA purification kit (217084, Qiagen). Briefly, 20-25 spheroids were mixed with Qiazol before being vortexed briefly and incubated at room temperature (rt) for 5 min. Samples were frozen at −80°C and RNA was extracted according to the manufacturer’s instructions. RNA was eluted with 14 μL of RNase-free water, and the purity and concentration were measured using a NanoDrop. For cDNA synthesis, the Maxima First Strand cDNA Synthesis kit was used (K1641, ThermoFisher). A dsDNA wipeout step was completed by combining dsDNase and 50 ng of template RNA. Gene expression was measured using RT-qPCR with the 2X SYBR Green mix (Qiagen) and gene primers of interest (Supplementary Table S7). Primers were designed to span exon-exon junctions (Integrated DNA Technologies; Coralville, IA, USA). The SYBR master mix included primers at a concentration of 500 n m and a cDNA concentration of 1 ng RNA per well. RT-qPCR was then performed using a Biorad CFX Opus 96 Real-time PCR system. Ribosomal protein S13 (RPS-13) was used as the reference gene as this gene has been shown to be stably expressed in experiments with alcohol as a variable.^11^ The 2^−DDCt^ method was used for data analysis, and the results of target genes were expressed as fold change relative to the control group.^53^

The following markers of lipid homeostasis were assessed. Fatty acid synthesis and its regulation [CHREBP, sterol regulatory element binding protein 1 (SREBP1), fatty acid synthase (FAS), acetyl-CoA carboxylase (ACC), stearoyl-CoA desaturase 1 (SCD1), and peroxisome proliferator activating receptor gamma (PPARG)], triglyceride storage [perilipins 1, 2, 3, 4, and 5 (PLIN1-5)], fatty acid uptake and intracellular transport [cluster of differentiation 36 (CD36) and fatty acid binding protein 1 (FABP1)], triglyceride breakdown and fatty acid mobilization [hepatic lipase (HL) and adipose triglyceride lipase (ATGL)], triglyceride synthesis [glycerol-3-phosphate acyltransferase, mitochondrial (GPAT1), diacylglycerol O-acyltransferase 1 (DGAT1) and stearoyl-Coenzyme A desaturase 1 (SCD1)], and triglyceride export [apolipoprotein B (APOB)] (Figure 3C).

Markers of regulation of beta-oxidation [PPARA, PPARD, Carnitine/Acylcarnitine Translocase (SLC25A20), Medium-Chain Acyl-CoA Dehydrogenase (ACADM), and carnitine palmitoyl transferase 1 (CPT1)] (Figure 3D, Supplementary Table S7); mitochondrial master regulators (PGC1A and PGC1B), and glycolysis rate-limiting enzyme [pyruvate kinase liver/red blood cell type (PKLR)] were assessed (Figures 5-6). Catalase, co-enzyme Q10 binding protein A (COQ10A), glutathione peroxidases 1 and 4 (GPX1, GPX4), glutathione synthetase (GSS), glutathione reductase (GSR), and superoxide dismutase 2 (SOD2) were assessed as markers of oxidative stress (Figure 4).

### Triglyceride Spheroid and Supernatant Quantification

Triglyceride content in each spheroid was quantified at endpoint using the Triglyceride-Glo™ Assay (J3160, Promega). Briefly, spheroids were washed with 1X PBS pH 7.4 before being transferred into glycerol lysis solution with lipase. Glycerol detection reagent was then incubated with the spheroids at rt for 1 h before reading luminescence. Glycerol content was measured using a standard curve. A no-lipase glycerol standard curve and no-lipase samples were generated to determine background glycerol in the sample. Background glycerol was quantified and subtracted from the lipase-containing samples for each group. As the background signal was negligible and did not alter the resulting relationships between different treatment groups, only the lipase-containing samples were used to determine the amount of triglyceride per sample.

To quantify supernatant triglyceride concentration, the same kit and protocol were used with the modifications described below. Supernatant was collected, pooled, and stored as described above (see albumin secretion quantification). Because fetal bovine serum is in the media and contains triglycerides, control, ethanol, sugar-fat, and ethanol sugar-fat media were used as controls to determine background triglyceride content. Twenty microliters of supernatant or media was mixed with glycerol lysis solution with lipase and a standard curve was used as described above. Net supernatant triglyceride concentration was calculated as sample triglyceride minus media triglyceride content. The ratio between pooled (average of 10 wells) supernatant and spheroid triglyceride concentration for 3 independent experiments per group was then calculated and averaged.

### Lipid Staining and Imaging

Spheroids were washed, fixed in Z-fix for 2 h, washed, and stored at 4°C. Five to ten spheroids per experimental group were stained for 30 min with DRAQ5 (dsDNA stain) and BODIPY 493/503 to detect neutral lipids as described elsewhere.^54^ After staining, spheroids were washed with 1X PBS pH 7.4 twice in the dark and imaged using a Leica confocal microscope. Z-stacks were approximately 35 μm deep. Maximal projections of confocal images were quantified by using the Adobe Photoshop color selection tool, and the integrated density of BODIPY staining was measured together with the total area of the DRAQ5 stain. BODIPY staining of each spheroid was normalized to DRAQ5 stain-derived spheroid area. Images of 3-6 spheroids per treatment group were analyzed across independent experiments.

### H_2_O_2_ Production Quantification

H_2_O_2_ was quantified using Amplex Red with an adapted method as described by the manufacturer (A22188, ThermoFisher) and detailed here^55^. Briefly, spheroids were washed two times in Krebs buffer, and spheroids (10 per well) from each experimental group were transferred to 50 μL of Krebs buffer in triplicate and incubated at 37°C. Positive (5 μm H_2_O_2_ in Krebs buffer) and negative (Krebs buffer) controls were prepared and run on each plate. Prepared Amplex Red activity reagent was added, and the plate was briefly mixed. The kinetic reaction was allowed to occur at 37°C for over an h, and fluorescence at 590 nm was measured every 300 s. Fluorescence versus time plots were generated, the slopes of each kinetic plot were determined, and the groups were compared relative to the control.

### ATP Quantification

ATP content was assessed using the Promega CellTiter-Glo^®^ 3D Cell Viability Assay according to manufacturer instructions with slight modifications (G9681). Individual spheroids were transferred to a white assay plate, along with 40 μL of media, and then mixed with the CellTiter Glo assay lysis buffer. Spheroids were mixed for 5 min and allowed to stabilize. Luminescence was measured using Cytation 1 (Biotek Instruments, Agilent Technologies).

### Mito Stress Test

Spheroid plates were coated with poly-D-lysine hydrobromide, washed with water, and allowed to dry before adding assay media and incubating at 37°C. Five spheroids were then transferred per well with 3-6 replicates per experimental group. The Agilent Mito Stress Test (Agilent, 103015-100) was performed using the following inhibitor concentrations: 2.5 μm oligomycin, 2 μm FCCP, and 1 μm Rotenone/Antimycin A. Brightfield and fluorescent images of Hoechst 33342-stained spheroids were taken to visualize spheroids. Spheroid area was averaged for 10 spheroids across groups and wells for each independent experiment using the Hoechst 33342-stained spheroid images using the same method described for DRAQ5 area quantification. Data were corrected to oxygen consumption rate (OCR) (pmol/min) or extracellular acidification rate (ECAR) (mpH/min) per spheroid and normalized to average spheroid area (mm^2^) for each treatment group. The metabolic phenotype and potential plot was generated as described by the manufacturer (Agilent) for adaptation to the Mito Stress Test, with the baseline phenotype being determined from the last measurement before oligomycin injection and the stressed phenotype being determined from the maximal measurement after oligomycin injection.

### Statistical Analysis

For comparisons between the core four groups of interest (control, ethanol, sugar-fat, and ethanol sugar-fat), two-way ANOVA was used with Fisher’s LSD post-hoc pairwise test when *n* ≤ 5 or Tukey’s correction when *n* > 5. Post-hoc pairwise comparisons were conducted despite nonsignificant interactions to explore specific group differences. For comparisons between all groups (factors: sugar, fat, and/or ethanol), three-way ANOVA was used with Fisher’s LSD post-hoc pairwise test when *n* ≤ 5 or Tukey’s correction when *n* > 5. Rates of kinetic reactions were analyzed using an adaptation of ANCOVA wherein the slopes of kinetic plots were analyzed using two-way ANOVA. All analyses were performed using GraphPad Prism 10.4.1. Statistical results are discussed as main effects or interactions of the treatments described (ethanol, sugar, and fat), and post-hoc pairwise comparisons are denoted as the specific treatment groups (C, E, SF, ESF, F, S, EF, or ES). Statistical results are discussed throughout the text and tabulated in Supplementary Tables S1-S6.

## Results

### Spheroid Culture and Hepatocyte Characterization

**Figure 1.**
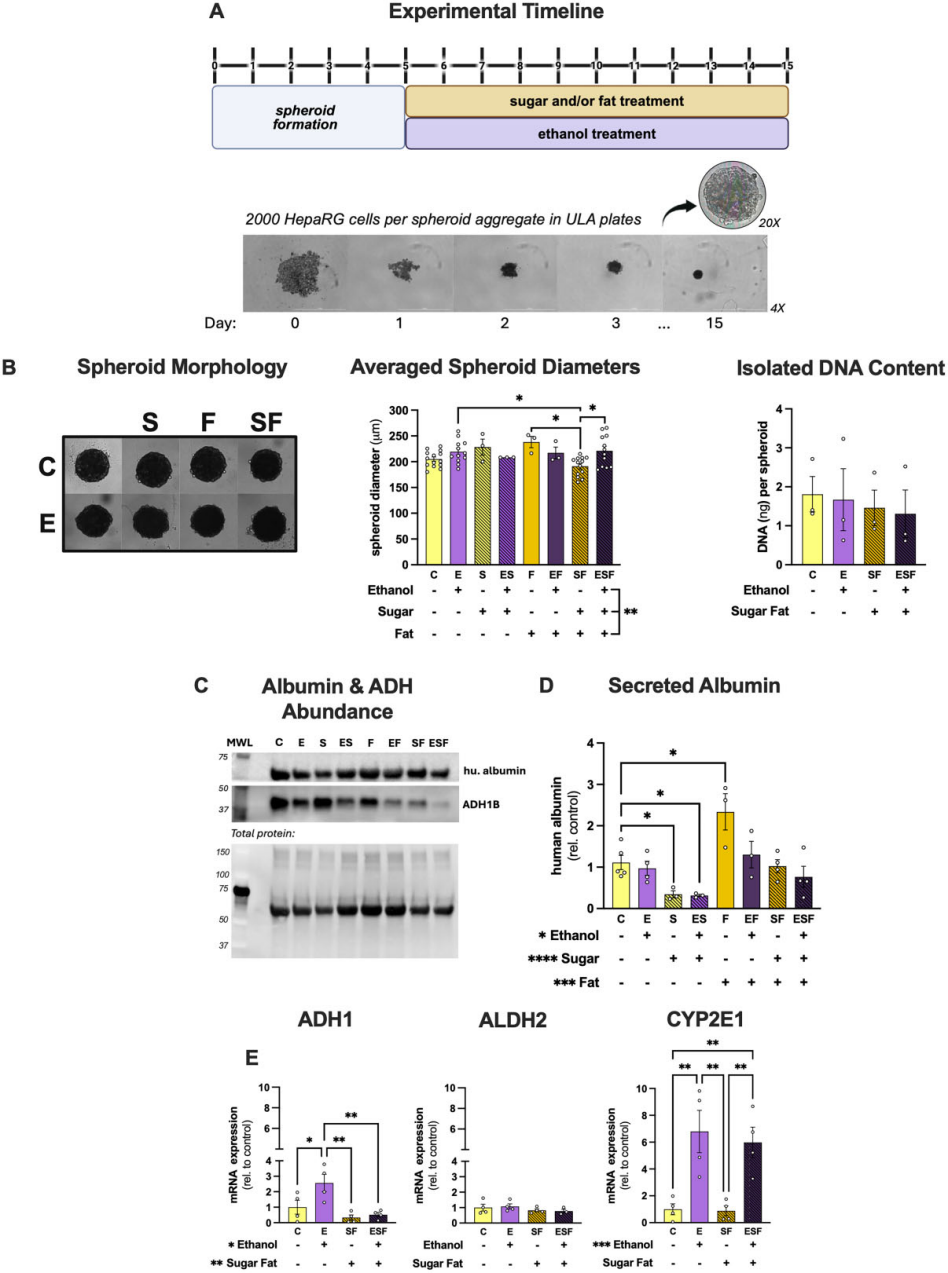
Experimental design and model characterization. (A) Spheroid culture example timeline and treatment paradigm. Created in https://BioRender.com. (B) Representative images of spheroid morphology from each treatment group at endpoint (10 d of treatment), averaged spheroid diameters, and isolated DNA content per spheroid. Each point represents averaged diameters or DNA recovered from independent experiments. Significant post-hoc comparisons are displayed for all comparisons. (C) Representative Western blot of albumin and alcohol dehydrogenase 1 (ADH1) protein abundance from 5 pooled spheroids per group. (D) Secreted human albumin content at endpoint. Significant post-hoc comparisons are displayed only for those between the control and treatments. (E) Normalized mRNA expression of ADH1, aldehyde dehydrogenase 2 (ALDH2), and cytochrome P450 enzyme 2E1 (CYP2E1) relative to control. Two-way or three-way ANOVA was used with post-hoc comparisons. Main effects are shown at the bottom of each bar graph and post-hoc comparisons are shown at the top of each graph. *P < 0.05, **P < 0.01, ***P < 0.001, and ****P < 0.0001.

Spheroids from 2000 cells per well were allowed to form for at least 3 d before treating with either control (C), ethanol (E), sugar (S), fat (F), ethanol-sugar (ES), ethanol-fat (EF), sugar-fat (SF), or ethanol-sugar-fat (ESF) conditions for 10 d (Figure 1A). There was an interaction between sugar, fat, and ethanol on spheroid diameter (*P* = 0.0031), with post-hoc analysis showing SF spheroids (191 ± 5μm) being smaller than F (238 ± 11 μm, *P* = 0.0254), E (219 ± 6 μm, *P* = 0.0346), and ESF (221 ± 9 μm, *P* = 0.0197) spheroids (Figure 1B; Supplementary Table S1).

The spheroids expressed the hepatocyte marker, albumin (Figure 1C), and alcohol metabolizing enzyme alcohol dehydrogenase 1 (ADH1B) (Figure 1C). There were main effects of both ethanol and sugar to decrease (*P* < 0.0001 and *P* = 0.0004, respectively) and fat to increase (*P* = 0.03) albumin secretion. Post-hoc comparisons showed that relative to the C group, the S and ES groups decreased, and the F group increased albumin secretion (Figure 1D). E, EF, SF, and ESF groups had no significant effect on albumin secretion compared to the C group (Figure 1D; Supplementary Table S1). There was a main effect of ethanol to increase ADH1 gene expression, whereas sugar-fat decreased ADH1 gene expression (Figure 1E). There was no significant effect on ALDH2 expression (Figure 1E). There was a main effect of ethanol to increase CYP2E1 gene expression, and sugar-fat had no significant effect on CYP2E1 expression (Figure 1E; Supplementary Table S1).

### Steatotic Phenotype

**Figure 2.**
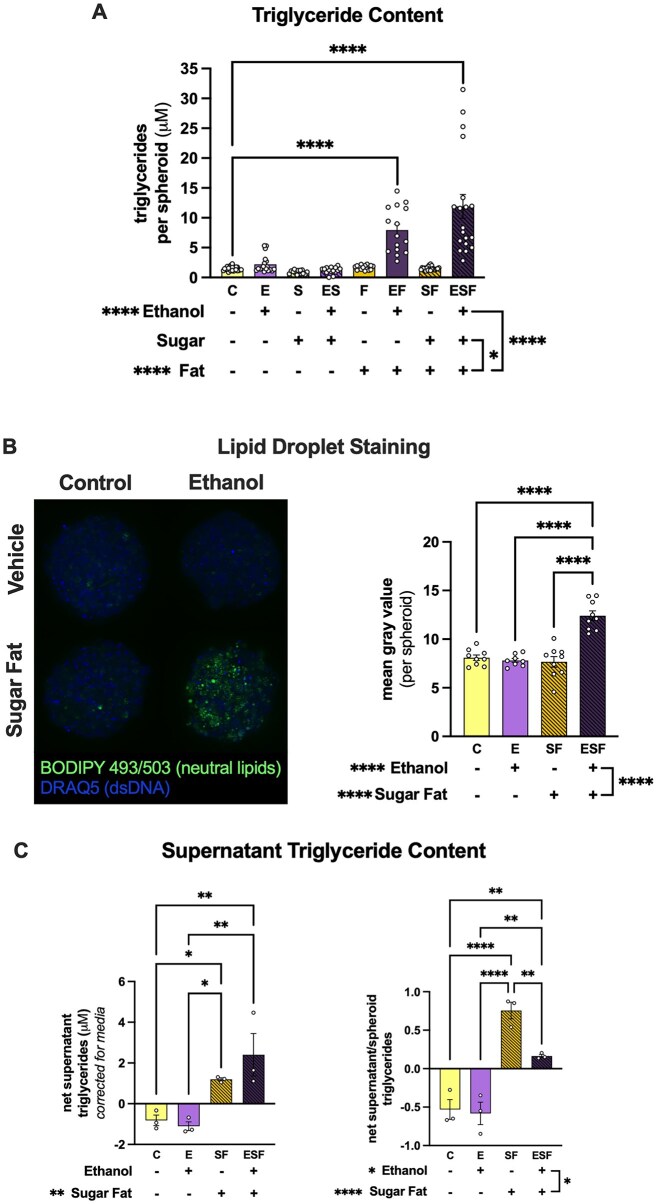
Ethanol and fat drive the increase in lipid content in HepaRG spheroids. (A) Triglyceride concentration per spheroid after 10 d of ethanol, sugars, and/or fats. Each point represents a single spheroid across at least 3 independent experiments. Only post-hoc comparisons between the treatment groups and control are displayed. (B) Lipid droplet staining in ethanol and combined metabolic stressor (sugar-fat) groups. A representative panel of maximal projections from confocal fluorescent imaging is shown with BODIPY staining neutral lipids and DRAQ5 staining nuclei. Lipid droplet staining in spheroids from each experimental group was compared using mean gray value per spheroid. (C) Net supernatant triglyceride concentration and supernatant to intracellular triglyceride ratio. Each point represents averages from matched independent experiments. Statistical differences in triglyceride content were assessed using three-way ANOVA and that of lipid droplet staining and net supernatant triglyceride content with two-way ANOVAs. Main effects are shown at the bottom of each bar graph and post-hoc comparisons are shown between groups at the top of each graph. *P < 0.05, **P < 0.01, ***P < 0.001, and ****P < 0.0001.

Lipid content was determined by quantifying triglyceride content per spheroid and by neutral lipid staining (Figure 2 and Supplementary Figures S1-S2). There were significant interactions between sugar and fat (*P* = 0.0232) as well as fat and ethanol (*P* < 0.0001) on triglyceride content (Figure 2A). There was a non-significant 3-way interaction between ethanol, fat, and sugar (*P* = 0.0517). There was a significant increase in triglyceride content in EF and ESF groups compared to the C group and their respective controls (Supplementary Table S2; Figure 2A). There were no significant differences in triglyceride content between C and E groups, nor between C and S, ES, F, or SF groups (Supplementary Table S2; Figure 2A).

As the focus of this work was to understand the combined effect of ethanol and metabolic stressors (ie, sugars and fats), lipid droplet content was assessed in the four main treatment groups: C, E, SF, and ESF by BODIPY staining. Our results showed an interaction between ethanol and metabolic stressors (sugar and fat together) (*P* < 0.0001; Figure 2B). There was a significant increase in BODIPY staining in the ESF group compared to all other groups (Figure 2B).

To assess whether triglyceride secretion may be affected by ethanol and metabolic stressors, we determined triglyceride content in the supernatant at endpoint. After correcting for triglyceride concentration in the culture media, which contains fetal bovine serum, results showed a main effect of sugar-fat treatment on net supernatant triglycerides (Figure 2C, Supplementary Table S2). Compared to the C group, both SF (*P* = 0.0321) and ESF (*P* = 0.0032) groups had increased net supernatant triglycerides (Figure 2C). To ascertain whether there was a relationship between the amount of triglycerides intracellularly and in the supernatant, we normalized net supernatant triglyceride content to spheroid triglyceride content from matched independent experiments (Figure 2C). There were main effects of both ethanol (*P* = 0.0203) and sugar-fat (*P* < 0.0001) as well as an interaction between the factors (*P* = 0.0411) on the ratio between supernatant and intracellular triglyceride content (Figure 2C). While both C and E groups maintained a lower triglyceride ratio compared to sugar-fat groups (Figure 2C, Supplementary Table S2), the ESF group had a lower triglyceride ratio compared to the SF group (*P* = 0.0055; Figure 2C).

### Lipid Homeostasis

**Figure 3.**
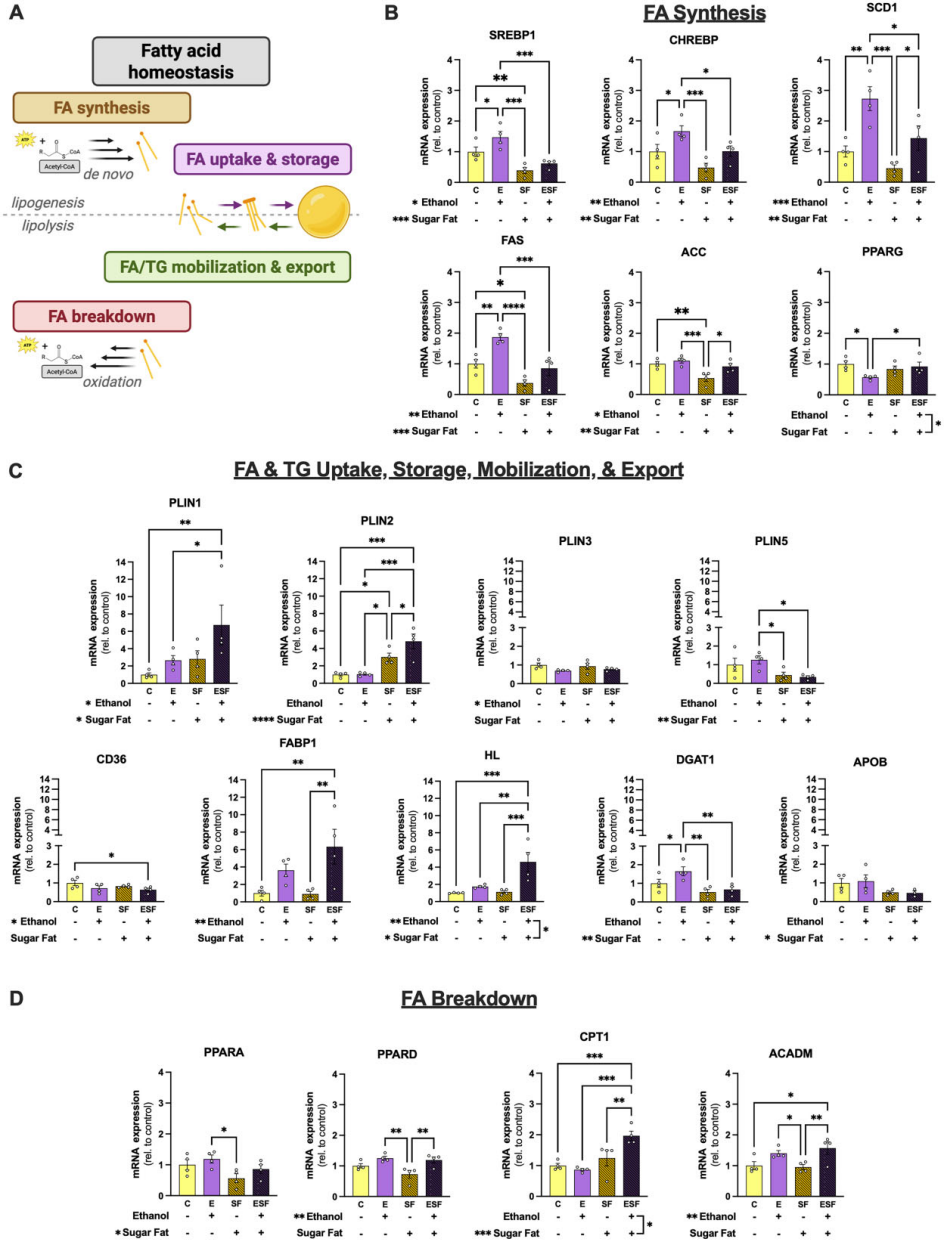
Ethanol and metabolic stressors independently and in combination impact gene expression of proteins central to lipid metabolism and storage. (A) Schematic illustrating the major regulatory pathways of fatty acid and triglyceride homeostasis. Created in https://BioRender.com. (B) Gene expression of fatty acid synthesis regulators (SREBP1, CHREBP, and PPARG) and major enzymes (FAS, ACC, and SCD1) that were significantly different between groups. (C) Gene expression of fatty acid uptake proteins (CD36, FABP1, and HL), lipid storage and dynamics regulator proteins (PLIN1-3,5 and DGAT1), and a triglyceride export protein (APOB) that were significantly different between groups. (D) Gene expression of fatty acid breakdown proteins and enzymes (PPARA, PPARD, CPT1, and ACADM) that were significantly different between groups. Each point represents 20-25 pooled spheroids from 4 independent experiments. Two-way ANOVA with Fisher’s LSD post-hoc analysis was used for analysis. *P < 0.05, **P < 0.01, ***P < 0.001, and ****P < 0.0001.

To identify potential pathways that contribute to lipid accumulation in the presence of ethanol and metabolic stressors, gene expression of major targets involved in fatty acid and triglyceride homeostasis was determined (Figure 3; Supplementary Figure S3). There were main effects of ethanol to increase (all *P* < 0.05) and sugar-fat to decrease CHREBP, SHREBP1, FAS, ACC, and SCD1 gene expression (all *P* < 0.01; Figure 3B). There was an interaction between ethanol and sugar-fat on PPARG expression (*P* = 0.0285), with decreased expression in the E group compared to C and increased expression in the ESF group compared to E (Figure 3B, Supplementary Table S3).

There were main effects of ethanol and sugar-fat to increase PLIN1 expression (both *P* < 0.05), and PLIN1 was increased in ESF compared to both the C and E groups (Figure 3C). There was a main effect of sugar-fat to increase PLIN2 (*P* < 0.0001; Figure 3C). PLIN2 expression was increased in SF and ESF groups compared to the C group, and there was a significant increase in the ESF group compared to SF and E groups (Figure 3C). There was a main effect of ethanol to decrease PLIN3 (*P* = 0.0371), and a main effect of sugar-fat to decrease PLIN5 (*P* = 0.0063; Figure 3C). There was no significant effect of these factors on PLIN4 (Supplementary Figure S3).

There was a main effect of ethanol to decrease CD36 (*P* = 0.0487), and CD36 in the ESF group was significantly decreased compared to the C group (Figure 3C). There was a main effect of ethanol to increase FABP1 (*P* = 0.0029), with significant differences between ESF compared to the C and SF groups (Figure 3C). There were main effects of both ethanol and sugar-fat (*P* = 0.0027 and *P* = 0.0209, respectively) and an interaction between these factors on HL (*P* = 0.0317). HL expression was increased in the ESF group compared to all other groups (Figure 3C). Ethanol and sugar-fat had no significant effect on ATGL gene expression (Supplementary Figure S3). While there was no effect of ethanol or sugar-fat on GPAT1 expression, there was a main effect of sugar-fat to decrease DGAT1 (*P* = 0.0027; Supplementary Figure S3 and Figure 3C). There were main effects of ethanol to increase and sugar-fat to decrease SCD1 (Figure 3B). There was a main effect of sugar-fat to decrease APOB expression (*P* = 0.0319; Figure 3C)

There was a main effect of sugar-fat to decrease PPARA (*P* = 0.0053), where the SF group decreased expression compared to the C and E groups (Figure 3D). There was a main effect of ethanol to increase PPARD (*P* = 0.0024), and the ESF group increased PPARD expression compared to the SF group (Figure 3D). There was a main effect of ethanol to increase ACADM expression (*P* = 0.0028) and a significant increase in the ESF group expression compared to both C and SF groups (Figure 3D). There was a main effect of sugar-fat on CPT1 (*P* = 0.0009) and an interaction between the factors (*P* = 0.0166; Figure 3D). Compared to C, E, and SF groups, ESF increased CPT1 gene expression (Figure 3D). There was no significant effect of ethanol or sugar-fat on SLC25A20 gene expression (Supplementary Figure S3).

### Oxidative Stress

**Figure 4.**
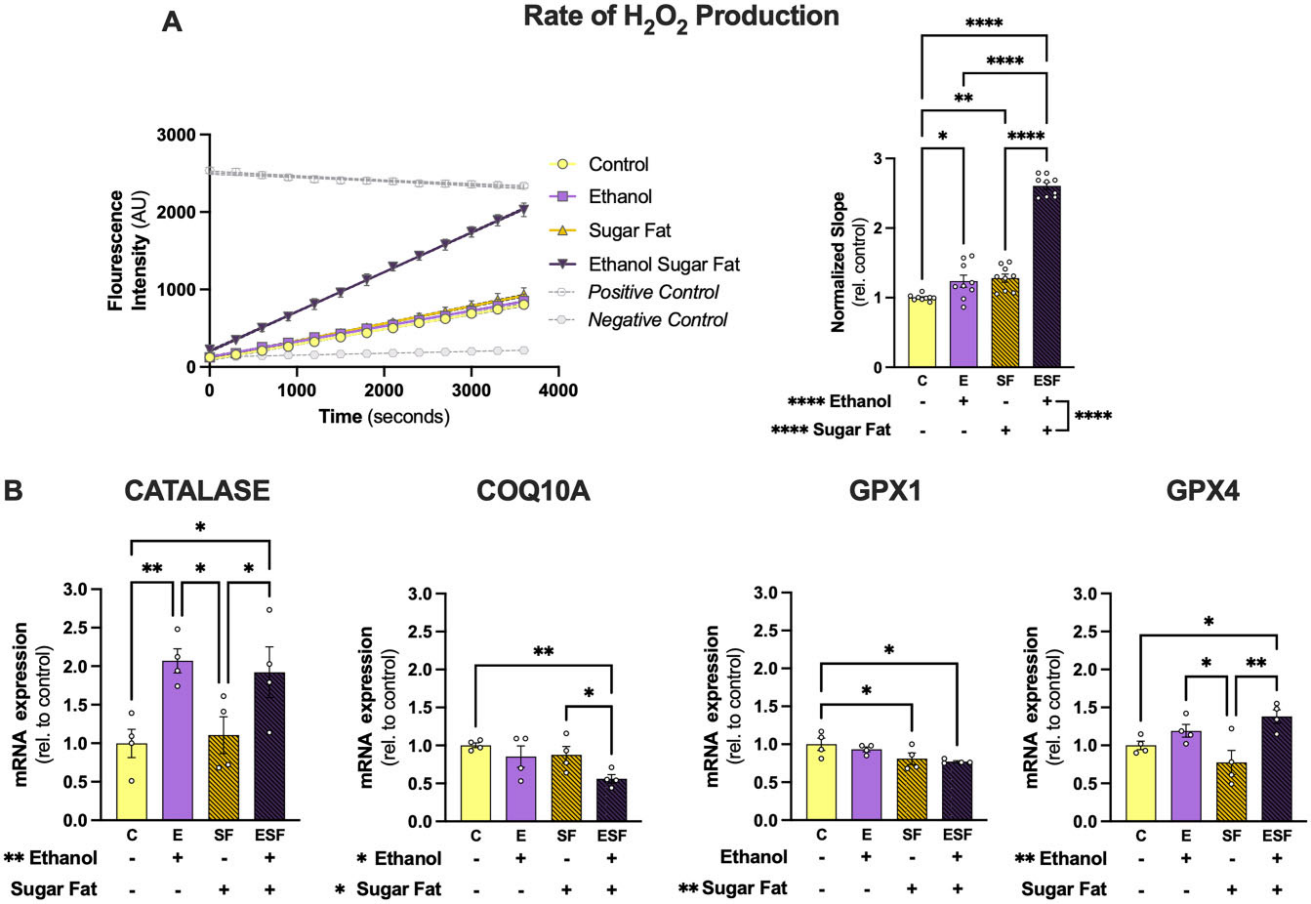
Ethanol and metabolic stressors synergistically increase oxidative stress in HepaRG spheroids. (A) Rate of H_2_O_2_ production measured using an Amplex Red assay in 10 pooled, intact HepaRG spheroids. Results are shown from 3 independent experiments. A representative trace of fluorescent intensity over time in triplicate is shown on the left with positive (5 μm H_2_O_2_ in Kreb’s buffer) and negative (Kreb’s buffer) controls displayed, and the rates of H_2_O_2_ production normalized to control values for each independent assay is shown on the right. (B) Gene expression of markers of oxidative stress regulation. Catalase, Co-enzyme Q10 binding protein A (COQ10A), and glutathione peroxidases 1 (GPX1) and 4 (GPX4). Two-way ANOVA with either Tukey’s post-hoc (for Amplex Red measures; *n* > 5 per group) or Fisher’s LSD post-hoc (gene expression; *n* < 5) comparisons were used for analysis. *P < 0.05, **P < 0.01, ***P < 0.001, and ****P < 0.0001.

Amplex Red was used to assess the generation of H_2_O_2_ over time in the four main groups (Figure 4A). There were main effects of ethanol and sugar-fat and an interaction on the rate of H_2_O_2_ production relative to control (all *P* < 0.0001). E and SF groups showed significant increases in the normalized rate of H_2_O_2_ production relative to control (Figure 4A). The ESF group had an increased rate of H_2_O_2_ production compared to C, E, and SF groups (Figure 4A).

Gene expression of ROS regulators was determined (Figure 4B). Ethanol increased catalase expression (main effect: *P* = 0.0017) in the presence and absence of sugar-fat compared to respective controls (Figure 4B). Sugar-fat decreased GPX1 expression (main effect: *P* = 0.0099), and ethanol increased GPX4 expression (main effect: *P* = 0.0024; Figure 4B). SF and ESF groups had decreased GPX1 expression compared to the C group (Figure 4C). The ESF group had increased GPX4 expression compared to both C and SF groups (Figure 4B). There were main effects of ethanol and sugar-fat to decrease expression of COQ10A (*P* = 0.0319 and *P* = 0.0488, respectively). There was a significant decrease in COQ10A expression in the ESF group compared to both C and SF groups (Figure 4B). There was no effect of ethanol or sugar-fat on GSS and GSR gene expression (Supplementary Figure S4).

### Bioenergetic Function and Mitochondrial Regulation

**Figure 5.**
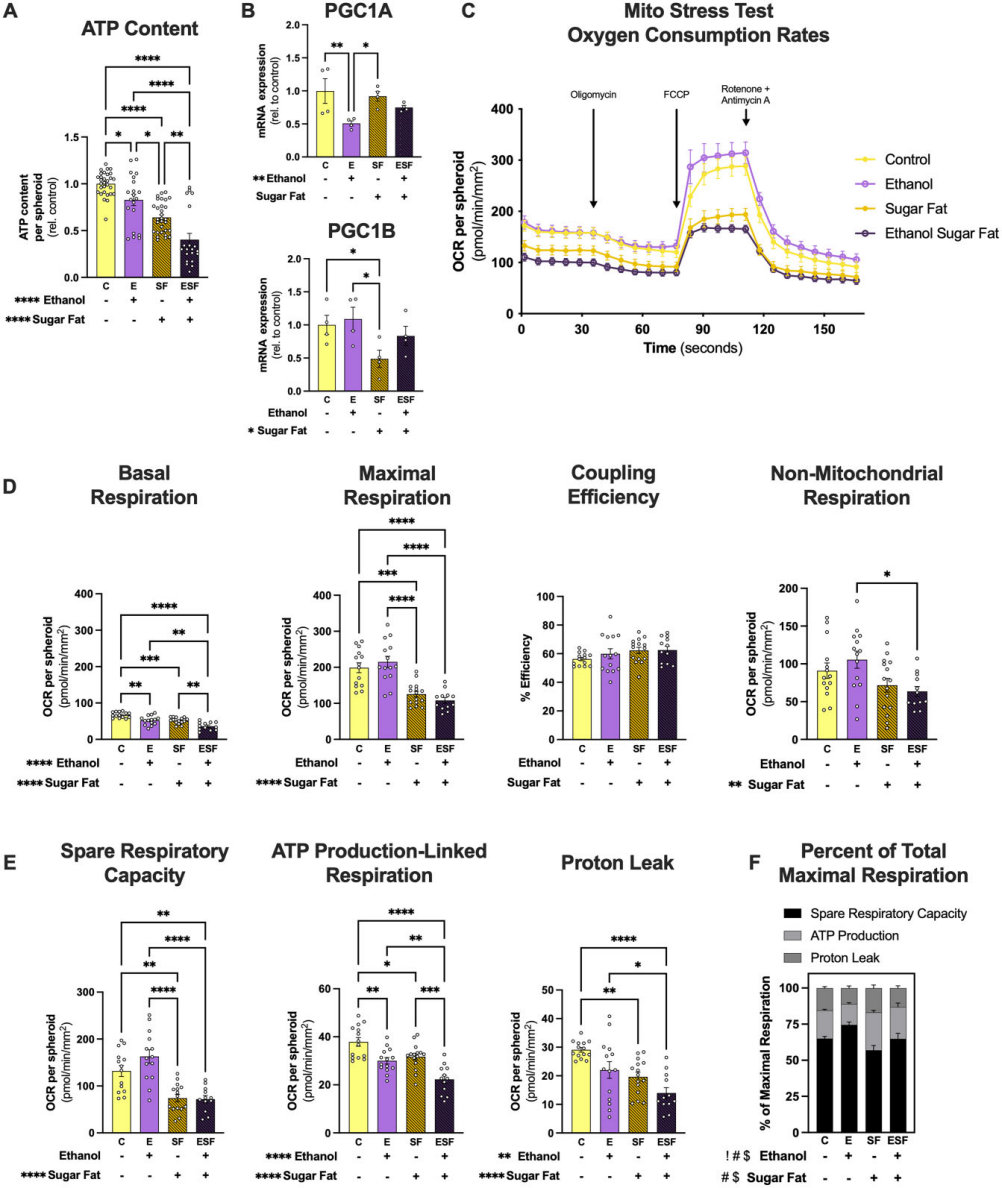
Ethanol and metabolic stressors in combination additively decrease ATP production in HepaRG spheroids. (A) ATP content after 10 d of treatment with ethanol and metabolic stressors. Each point represents a single spheroid across at least four independent experiments. (B) Gene expression of master regulators of mitochondrial homeostasis (PGC1A and PGC1B) that were significantly different between groups. (C) Average OCRs across treatment groups from the Mito Stress Test. C (*n* = 14), E (*n* = 14), SF (*n* = 15), and ESF (*n* = 12), where each *n* represents OCR corrected to pmol/min/spheroid and normalized to average spheroid area (mm^2^) across 3 independent experiments (mean ± SEM). (D) Basal, maximal, and non-mitochondrial respiration and coupling efficiency. (E) Spare respiratory capacity, ATP-production-linked respiration, and proton leak from ethanol and metabolic stressor-treated spheroids. (F) Normalized (to 100% of each individual replicate) maximal respiration stratified by the % attributable to spare respiratory capacity, ATP production, and proton leak. # represents a significant main effect for % spare respiratory capacity, $ represents a significant main effect for % ATP-production-linked respiration, and ! represents a significant main effect of proton leak. Two-way ANOVA with either Tukey’s post-hoc (for ATP content and Mito Stress Test measures; *n* > 5 per group) or Fisher’s LSD post-hoc (gene expression, *n* < 5 per group) comparisons were used for analysis. *P < 0.05, **P < 0.01, ***P < 0.001, and ****P < 0.0001.

There were main effects of ethanol and sugar-fat (*P* < 0.0001) to decrease ATP content (Figure 5A). ATP content was decreased in E, SF, and ESF groups compared to the C group (Figure 5A).

There were main effects of ethanol to decrease PGC1A gene expression (*P* = 0.0077) and sugar-fat to decrease PGC1B gene expression (*P* = 0.0257; Figure 5B). PGC1A expression was decreased in the E group compared to the C and SF groups (Figure 5B). The SF group had decreased PGC1B expression compared to both C and E groups (Figure 5B).

**Figure 6.**
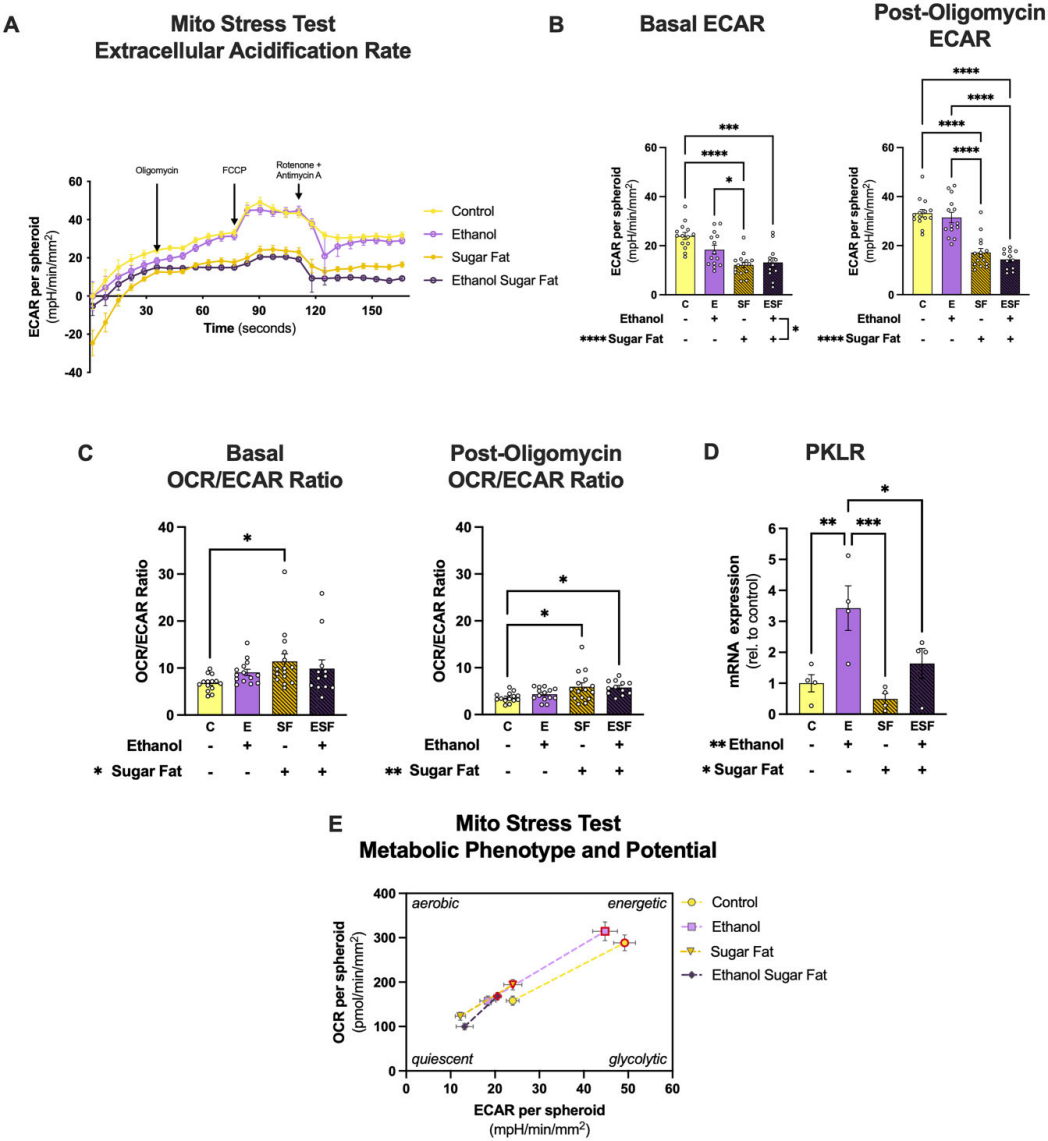
Metabolic stressors decrease ECAR and alter the metabolic phenotype in HepaRG spheroids. (A) Averaged (mean ± SEM) ECAR traces from the Mito Stress Test. C (*n* = 14), E (*n* = 14), SF (*n* = 15), and ESF (*n* = 12), where each n represents ECAR corrected to mpH/min/spheroid and normalized to average spheroid area (mm^2^) across 3 independent experiments (mean ± SEM). (B) Basal and post-oligomycin ECAR. (C) Basal and post-oligomycin OCR/ECAR ratios. (D) Gene expression of pyruvate kinase (PKLR), a rate limiting enzyme of glycolysis. (E) Mito Stress Test Metabolic Phenotype and Potential map generated from determining average baseline (OCR and ECAR just before oligomycin injection) and stressed (maximal OCR and ECAR after oligomycin and FCCP injections) states. Average baseline states are indicated by grey boarders, and stressed states by points with red boarders. Two-way ANOVA with either Tukey’s post-hoc (for Mito Stress Test measures; *n* > 5 per group) or Fisher’s LSD post-hoc (gene expression; *n* < 5 per group) comparisons were used for analysis. *P < 0.05, **P < 0.01, ***P < 0.001, and ****P < 0.0001.

To assess real-time oxygen consumption and the contributions of ETC components to energy metabolism, the Seahorse Mito Stress Test was performed (Figure 5C-F and Figure 6). Basal OCR were decreased in E, SF, and ESF groups compared to C (Figure 5D). The basal OCR in the ESF group was also decreased compared to the E and SF groups (Figure 5D). There was a main effect of sugar-fat to decrease maximal respiration (*P* < 0.0001) and non-mitochondrial oxygen consumption (*P* < 0.0027), but there was no significant change in coupling efficiency between the groups (Figure 5D). There was a main effect of sugar-fat to decrease spare respiratory capacity (*P* < 0.0001; Figures 5E). There was an additive effect of ethanol and sugar-fat to decrease ATP production, with main effects of both factors individually (Figure 5E). There were main effects of ethanol and sugar-fat to decrease proton leak (*P* = 0.0021 and *P* < 0.0001, respectfully; Figure 5E). After normalizing maximal respiration to 100% for each replicate, the percentages of maximal respiration attributable to spare respiratory capacity, ATP-production-linked respiration, and proton leak were calculated (Figure 5F). There was a main effect of ethanol to increase (*P* = 0.0027) and sugar-fat to decrease (*P* = 0.0022) the % of maximal respiration attributable to spare respiratory capacity. Additionally, there were main effects of ethanol to decrease (*P* = 0.0035) and sugar-fat to increase (*P* < 0.0001) the % of maximal respiration attributable to ATP production (Figure 5F). There was a main effect of ethanol to decrease the % of maximal respiration attributable to proton leak (*P* = 0.0134; Figure 5F).

There was an interaction between ethanol and sugar-fat on basal ECAR as measured using the ECAR of the Mito Stress Test (*P* = 0.0428; Figure 6A-B). SF and ESF groups had lower basal ECAR compared to the C group (Figure 6B). There was a main effect of sugar-fat to decrease post-oligomycin ECAR (*P* < 0.0001; Figure 6B). There was a main effect of sugar-fat to increase both the basal and post-oligomycin OCR/ECAR ratios (Figure 6C). Further, there was an interaction between ethanol and sugar-fat on the difference between post-oligomycin and basal ECAR (*P* = 0.0025), with the ESF group being lower than both the C and E groups (*P* = 0.0001 and *P* < 0.0001, respectively; Supplementary Figure S5).

To compliment these estimates of glycolytic function, gene expression of pyruvate kinase (PKLR) was assessed (Figure 6D). Ethanol had a main effect to increase PKLR mRNA expression (*P* = 0.0023), whereas sugar-fat had a main effect to decrease PKLR expression (*P* = 0.0293; Figure 6D).

To qualitatively visualize the relationship between oxygen consumption and extracellular acidification in baseline and stressed states, a metabolic phenotype and potential map was generated using the Mito Stress Test results (Figure 6E). The SF and ESF groups were clustered in the quiescent quadrant in both the baseline and stressed states (Figure 6E).

## Discussion

The interplay between metabolic stress, excessive nutrient consumption, and alcohol misuse remains incompletely understood but is an essential focus for studying MetALD. In this study, we assessed the effects of alcohol in combination with metabolic stressors (high concentrations of sugars and fatty acids) on hepatocytes using a 3D spheroid model. We demonstrated synergistic effects of alcohol and metabolic stressors on lipid accumulation and oxidative stress, and additive effects on mitochondrial dysfunction.

Alcohol use in MetALD is associated with increased morbidity and mortality.^5,6,56,57^ However, the mechanisms through which alcohol interacts with the factors leading to MASLD are not clear. The development of MASLD has been linked to metabolic dysregulation including obesity, insulin resistance, hypertension, and dyslipidemia along with dietary and genetic factors.^58-61^ The relationship between these etiologies remains to be understood, but excessive consumption of calorie-dense diets and specific nutrients such as fructose and saturated fats shows positive associations with MASLD.^27,62^ Monosaccharides, such as glucose and fructose, and alcohol have been shown to contribute to steatosis through upregulation of DNL and triglyceride formation.^27,29,31^ In contrast, high fats generally decrease hepatic fatty acid synthesis as a negative feedback mechanism.^28,31^ Moreover, mitochondrial dysfunction and oxidative stress are additional key contributors to alcohol and metabolic-associated liver diseases.^36^ A separate phenomenon relevant to dual hit liver injury is the impact metabolic stressors can have on the detoxification capacity of the liver. Ethanol, a toxicant that is metabolized to acetaldehyde and then acetate, is largely metabolized in the liver, and thus alterations in alcohol or acetaldehyde metabolizing enzymes may lead to prolonged ethanol intoxication. Interestingly, in both humans with MASLD and rodent models of MASLD, several findings have shown impairment in ADH activity and protein expression and/or elevated fasting ethanol concentrations in studies focused on gut-derived ethanol production.^63,64^ Additionally, a link has been made to LPS-induced impairments in alcohol metabolism in a rodent model of liver injury and chronic alcohol consumption.^65^ These studies from MASLD patients and rodent models showing impairments in alcohol metabolism have been linked to pro-inflammatory signaling and oxidative stress.^64,65^ We observed similar decreases in the expression of ADH1 in HepaRG spheroids treated with sugars and fats, suggesting a possible direct role of sugars and fats on hepatocytes to also decrease the ability of hepatocytes to metabolize ethanol. Still, CYP2E1 was induced by ethanol in both the presence and absence of sugars and fats. Studies have shown HepaRG monolayer cultures and spheroids have the capacity to upregulate CYP2E1 and metabolize ethanol,^46,48^ and the present study is to our knowledge the first to assess the effects of metabolic stressors on alcohol metabolizing enzymes in HepaRG spheroids. Any change in the ability to metabolize ethanol due to metabolic stressors would be highly relevant to MetALD progression and should be further studied.^66,67^

One aim of this study was to explore interactions between ethanol and metabolic stressors on hepatocyte lipid accumulation. The findings in the present study align with the hypothesis that a synergistic relationship exists between alcohol and metabolic stressors, most notably fatty acids, to increase triglyceride and lipid droplet content in hepatocytes. This synergy has been identified in clinical measures of liver steatosis in people with SLD who report significant alcohol consumption compared to those who do not.^4,5,68^ Further, a rodent model of MetALD that uses 5 g/kg daily alcohol, weekly alcohol binges, and a MASH diet showed synergistic elevations in hepatic lipids.^21^ The present study complements rodent, human, and cell culture work by exploring hepatocyte-specific relationships between these metabolic stressors and alcohol on lipid dyshomeostasis. Interestingly, 10-d treatment with sugar and fat alone did not lead to increases in triglyceride content in HepaRG spheroids. However, using the same sugar and fat concentrations, our results showed increase lipid content in the presence of ethanol (ESF). We confirmed the ability of the metabolic stressor treatments to induce lipid accumulation by fatty acid overload in HepaRG spheroids. A serum-free induction medium was used and a higher oleic acid concentration (1 mM) as reported in studies validating steatosis induction.^47,69,70^ In addition, some studies that were focused on metabolic stressors prepared fatty acids using a high concentration (100 mM) of ethanol as a solvent.^71^ In order to maintain ethanol concentrations in culture studies, ethanol is added to the media and cells are placed in an ethanol-vapor incubator to mitigate evaporation. We confirmed that even the presence of ethanol in combination with fatty acids in the media without ethanol vapor incubation increases triglyceride content as well (Supplementary Figure S2). Taken together, our study design allowed for detecting synergy between ethanol, sugars, and fats and avoided ethanol as a confounder to studying hepatocyte responses in MetALD versus MASLD culture models.

Our data suggests a minor role of direct hepatocyte synergistic transcriptional upregulation of key DNL pathway regulators. While ethanol alone increased gene expression of fatty acid synthesis genes without inducing triglyceride accumulation, the ESF combination group did not increase expression of these genes compared to control, suggesting that the accumulation of lipids is associated with alternative pathways of lipid homeostasis. Additionally, metabolic stressors decreased expression of various regulators of fatty acid synthesis whereas the addition of ethanol to sugar and fats abolished this effect on gene expression of FAS, ACC, CHREBP, and SCD1. However, studies are warranted to assess the synergistic effects of alcohol and metabolic stressors on post-translational modifications of these regulators. Additionally, radiotracer experiments that allow for assessment of DNL through heavy carbon and hydrogen isotopes would further clarify the contribution of DNL to lipid accumulation observed in ESF-treated HepaRG spheroids. Alcohol and metabolic stressors have each been shown to disrupt hepatic fatty acid metabolism and storage.^10,72,73^ Interestingly, in HepaRG spheroids treated with ESF, upregulated genes included PLIN1, PLIN2, FABP1, and hepatic lipase. PLIN1 gene expression is upregulated in association with the development of larger lipid droplets in hepatocytes,^74,75^ which were observed in the ESF group. Larger droplets blunt lipase ability to break down triglycerides^74,75^ and may be partially responsible for the accumulation of lipids in the ESF group alone.

Export of triglycerides via VLDL is also impaired by ethanol^69,72^ and in progressed stages of MASLD,^76-78^ compounding the possible contributors to lipid overload by ESF. ESF treatment led to a lower supernatant to intracellular triglyceride ratio in HepaRG spheroids, suggesting that export may be impaired in this group, but more studies are needed to definitively determine whether the VLDL export process is impaired. Additionally, ESF-treated HepaRG spheroids had increased expression of hepatic lipase, which is shown to increase uptake of fatty acids from serum. ESF also increased FABP1, which binds to fatty acids intracellularly and allows for the regulation of genes involved in PPAR signaling pathways such as fatty acid storage and degradation.^79^ These changes may reflect the reason for increases in perilipin expression and CPT1 gene expression in the ESF group specifically, as they are downstream targets of the PPAR pathway.^79^ Taken together, transcriptional changes suggest increased lipid storage capacity and altered triglyceride dynamics due to direct effects of ethanol and metabolic stressors on hepatocytes compared to DNL. Whether these changes in gene expression preceded lipid accumulation, which only occurred in the ESF group in this study design, or if the gene expression changes are the result of excess lipid accumulation remains to be determined.

It is known that ethanol and metabolic stress increase oxidative stress individually, which can create an environment that perpetuates ROS generation and induces cellular damage.^18^ We assessed whether ethanol and metabolic stressors interact to further increase the ROS burden in HepaRG spheroids. Like the effect on lipid accumulation, ethanol and metabolic stressors synergize to increase ROS generation. As expected, CYP2E1 and catalase gene expression were induced by ethanol.^46^ While many antioxidant genes were unaffected by ethanol and/or metabolic stressors, COQ10A and GPX1 were decreased in the ESF group compared to control HepaRG spheroids. While determining the sources of excessive ROS was outside of the scope of this study, findings from other studies suggest that inadequate induction of antioxidant genes in the presence of elevated ROS production reflects a possible deficit in the ability of ESF-treated spheroids to respond to cellular stress.^80^ An additional source of excessive ROS is the electron transport chain, which could be dysregulated by alcohol and metabolic stressors.^81^

Mixed results from different models have contributed to a poorly understood relationship between ATP production capacity, mitochondrial respiration, and factors including ethanol and nutrient overload.^35,82^ Ethanol contributes to excess electron transfer through the generation of NADH but also leads to mitochondrial damage and decreased respiration capacity.^15^ In general, acute ethanol exposure has been linked to compensatory metabolic adaptations such as elevated NADH being alleviated through the electron transport chain and proton leakage. Changes in respiration due to nutrients are owed to more reduced cofactors, substrate flux through the TCA cycle, and enhanced uncoupling mechanisms.^37,82^ Additionally, nutrients such as sugars and fats show time- and dose-dependent effects on mitochondrial respiration and ATP production, with acute nutrient overload being associated with increased respiration and chronic exposure, or in MASH, decreased ATP and respiration.^37,47,71^ There is limited published work on how these factors interact to affect hepatocyte mitochondria after chronic exposure. Thus, we assessed bioenergetic function in HepaRG spheroids treated with the combination of ethanol, sugars, and fatty acids.

Results showed that in the ESF group, there was an additive decrease in ATP content and production compared to ethanol or sugar-fat treatments and a synergistic increase in lipid content. Like the present findings, in pro-obesogenic rats fed only a high-fat diet for 3 d, impairments in mitochondrial respiration preceded steatosis development.^82,83^ Moreover, in primary human hepatocyte 3D culture treated with fatty acids for 7 d showed concomitant bioenergetic impairment and lipid accumulation.^71^ Ethanol-treated spheroids showed small but significant changes in mitochondrial function, whereas sugar-fat and ESF-treated spheroids showed greater changes in maximal and basal respiration along with an additive decrease in ATP production, reflecting global impairment or suppression of mitochondrial respiration. Decreased global respiration can reflect decreased demand for ATP production,^84^ decreased mitochondrial membrane potential,^85,86^ and/or decreased healthy mitochondrial mass.^87-89^ Generally, increased substrate availability leads to an increase in mitochondrial function.^90^ We assessed whether the percentage of maximal respiration attributable to ATP production-linked respiration was increased in sugar-fat treated spheroids.^90^ Indeed, the % of maximal capacity attributable to ATP production in the sugar-fat groups was higher than respective controls, reflecting that while overall there is more mitochondrial impairment in SF groups and less ATP generated, the functional mitochondria can use the excess substrates provided more efficiently.^90,91^

While in this study we did not determine whether lipid accumulation or mitochondrial dysfunction occurs first, the additive impairment of ATP production by ESF may, in part, result from reduced adaptability of the ETC, a contributor to ROS generation.^91-93^ Although the Mitochondria Stress Test is not a comprehensive measure of ETC functionality, respirometry does allow for an overview of bioenergetic functionality and adaptability to stress.^93^ Specifically, the spare respiratory capacity, or mitochondrial reserve, is a measure that reflects the ability of the ETC to adapt in the presence of stressors or increased substrate to meet the ATP demands of the cell.^91,94^ Hepatocytes generally have a large spare respiratory capacity.^88,90,91,95^ When spare respiratory capacity is impaired, there could be an inadequate response to an increased demand for ATP resulting in cellular dysfunction or cell death.^91^ In HepaRG spheroids, spare respiratory capacity was decreased by sugars and fats, indicating that ethanol alone does not alter the ability of the hepatocyte to respond to stressors, but sugars and fats may directly limit the mitochondrial reserve. As isolated primary rat hepatocytes fed a chronic ethanol liquid diet show impaired respiratory capacity,^96^ the present findings suggest that the direct effects of ethanol may not be sufficient to impair maximal respiration and the mitochondrial reserve, but that the multicellular, multi-organ environment may greatly impact this parameter of respiration. Still, other studies have shown an increase in mitochondrial respiration in mouse models of chronic alcohol administration, wherein the findings were attributed in part to an adaptation to increased alcohol metabolism.^97^ Overall, the present study aligns with others in that ATP production was impaired by ethanol treatment and may reflect similar adaptations to ethanol metabolism that contribute to a lack of impairment in the spare respiratory capacity. Future studies using primary human hepatocyte spheroids treated with ethanol in vitro from individuals with and without histories of ALD may clarify the different responses of hepatocytes to ethanol that have been reported.

Ethanol and metabolic stressors independently elevated ROS, decreased ATP production, and altered gene expression of lipid metabolism markers. However, these changes did not produce significant alterations in intracellular triglycerides and lipid droplets in our experimental conditions. The combination of these stressors under these same conditions, however, increased lipid content and drove synergistic and additive effects on the cellular functions. Further, as would be expected with lipid overload, fatty acid oxidation regulators CPT1 and ACADM were upregulated by ESF.^98,99^ This upregulation did not lead to compensatory decreases in lipid content nor an increase in mitochondrial oxygen consumption and ATP production. Instead, ESF led to synergistic increases in H_2_O_2_ production indicating oxidative stress. Fatty acid oxidation generally increases in acute models of fatty acid overload, but over time decreases due to accumulation of mitochondrial damage.^37^ We postulate that the observed changes in gene expression may reflect increased fatty acid oxidation following lipid accumulation in ESF. This speculation is based on the lack of accumulation of lipids even in the absence of increased markers of mitochondrial fatty acid oxidation in the E and SF groups. Thus, lipid accumulation may precede fatty acid oxidation upregulation in this context. Synergistic increases in ROS by ESF may be due to oxidative stress associated with alcohol metabolism compounded by metabolic stressor-induced global suppression of mitochondrial bioenergetic function, leading to inadequate responses to cellular energetic demands. Nonetheless, while both lipid accumulation and bioenergetic impairment occur in response to combined ethanol and metabolic stressors, it remains unclear whether one precedes the other or whether a cyclical relationship exists. Future studies aimed at understanding the direct effects of alcohol, sugars, and fats alone and in combination on redox imbalances in hepatocytes will elucidate the relationships between oxidative stress, mitochondrial function, and lipid accumulation.

This study, though novel, is not without limitations including the use of only one hepatocyte progenitor cell line. However, HepaRG cells have been shown to better recapitulate a primary hepatocyte phenotype as compared to other cell lines such as HepG2 cells.^90^ Further, HepaRG cells are more resistant to overt lipid accumulation compared to primary rodent hepatocytes, allowing for the present study to identify the synergistic effects of ethanol and metabolic stressors at lower, biologically relevant levels of fatty acids and ethanol as compared to similar lipid-overload studies.^47^ Additionally, the use of 3D spheroids enhances the biological relevance of hepatocyte cultures as shown in numerous studies.^44-46^,^100-102^ Intriguingly, although only the Mito Stress Test was used in this study, sugar-fat treatment decreased ECAR as well in the spheroids, and the ESF-treated group appeared to have an impaired ability to support ATP production via glycolysis once ATP synthase was inhibited by oligomycin despite having an excess supply of sugars (Supplementary Figure S5). While the metabolic phenotype of sugar-fat treated spheroids remained closer to the quiescent quadrant as compared to control and ethanol-treated spheroids, which reflects relationships between both mitochondrial and glycolytic bioenergetic function, the Glycolysis Stress Test would be a more definitive measure of glycolytic function at baseline and in stressed states. Additionally, while there are some markers of alterations in overall mitochondrial regulation by ethanol, sugars, and fats due to changes in gene expression of PGC1s in HepaRG spheroids, this study did not assess effects of ethanol and metabolic stressors on mitochondrial dynamics, which are known to be disrupted in ALD and MASLD.^103,104^ As mitochondrial biogenesis, fusion, fission, and mitophagy are known to associate with impaired oxidative phosphorylation,^105^ whether some of the additive effects of ethanol and metabolic stressors are due to derangements in these processes should be elucidated.

**Figure 7.**
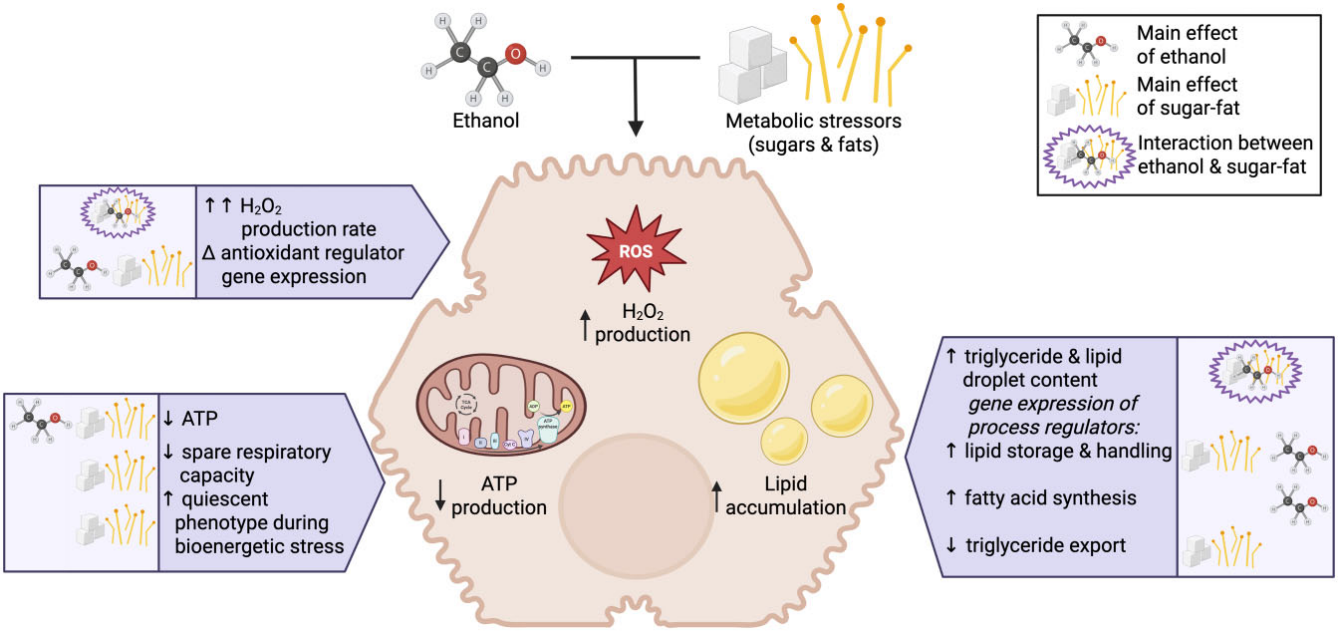
Summary of individual, synergistic, or additive effects of ethanol and metabolic stressors on hepatocyte-like spheroids. Ethanol and metabolic stressors synergistically increase ROS production and lipid content and additively decrease ATP production in hepatocytes. Sugar-fat treatment decreases spare respiratory capacity, displays a shift toward a quiescent bioenergetic phenotype, and decreases markers of triglyceride export. The combination of ethanol, sugars, and fats differentially alters gene expression of antioxidant regulators while increasing that of lipid storage regulators. Created in https://BioRender.com.

In summary, ethanol, sugars, and fats synergistically impact hepatocyte lipid homeostasis, oxidative stress regulation, and mitochondrial function (Figure 7). Future studies that mechanistically tease apart the salient markers of lipid dyshomeostasis and mitochondrial bioenergetic function will help answer questions regarding the relationship between hepatocyte lipid accumulation, ROS, and bioenergetics. Further, multi-cell organoids will aid our understanding of the whole-liver impacts of these hepatocyte-specific alterations.

## Supplementary Material

zqaf049_Supplemental_Files

## Data Availability

All data is available upon request to the corresponding author, Dr Liz Simon.
