# Supplementary material for: Alcohol and Metabolic Stress Synergize to Dysregulate Mitochondrial Health and Lipid Metabolism; Evidence from a Hepatocyte Spheroid Model
Source: Function (Oxf). 2025 Nov 3;6(6):zqaf049. doi: 10.1093/function/zqaf049 (PMC12605769; doi:10.1093/function/zqaf049)
Supplement: zqaf049_Supplemental_Files [file zqaf049_supplemental_files.zip › Supplemental_Information_10052025_revision_submitted.docx]

**Title:**

Alcohol and Metabolic Stress Synergize to Dysregulate Mitochondrial Health and Lipid Metabolism; Evidence from a Hepatocyte Spheroid Model

1. Supplementary Information
   1. Supplementary Figure 1. Time course lipid content.
   2. Supplementary Figure 2. Fatty acid overload.
   3. Supplementary Figure 3. Non-significant genes involved in lipid metabolism.
   4. Supplementary Figure 4. Mitochondrial function and ROS regulation.
   5. Supplementary Figure 5. Mito Stress Test post-oligomycin minus basal extracellular acidification rates.

**Supplementary Figures.**

| **A**  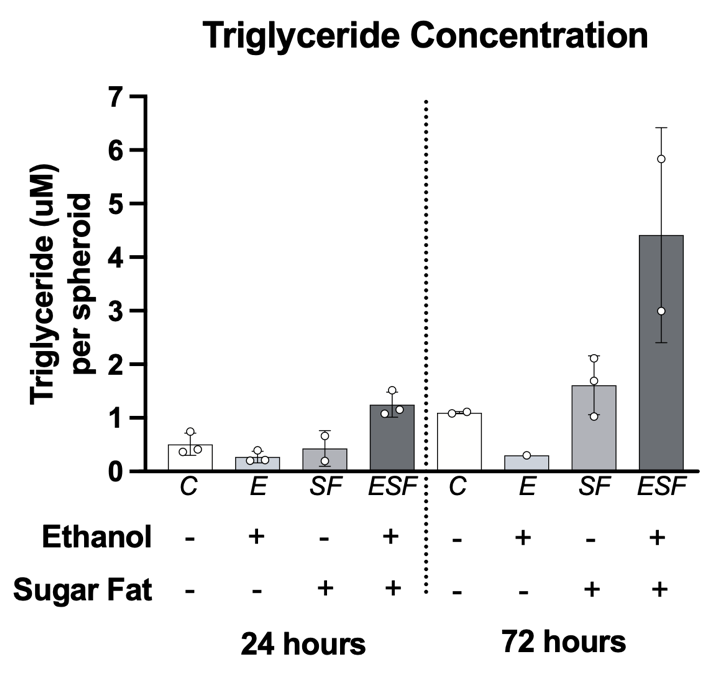 | **B**  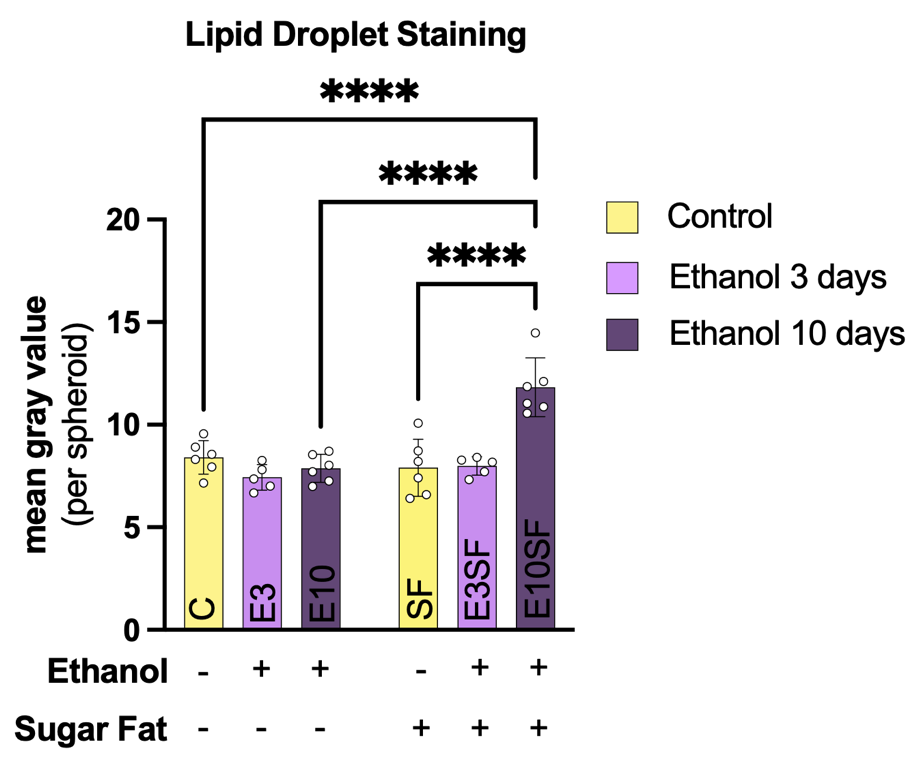 |
| --- | --- |
| **Supplementary Figure 1.** **Optimization of fatty acid overload experiment exposure time.** A) Quantified triglyceride content at 24 hours of treatment and 72 hours of treatment. Statistical analyses were not run due to the small sample size in some groups. B) BODIPY (neutral lipid) staining normalized to DRAQ5 (nuclear) staining area. Two-way ANOVA was used with Tukey’s post-hoc comparisons. ****p<0.0001. | |

| **A**  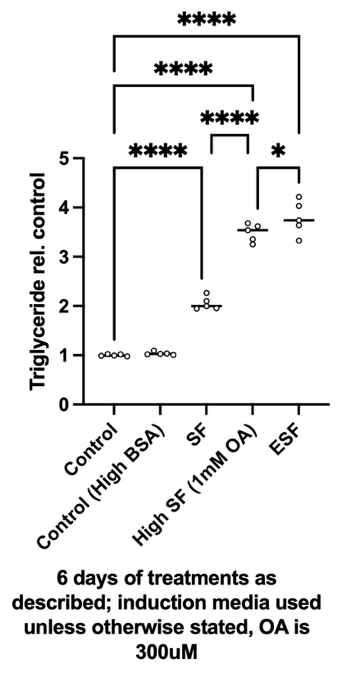 | **B**  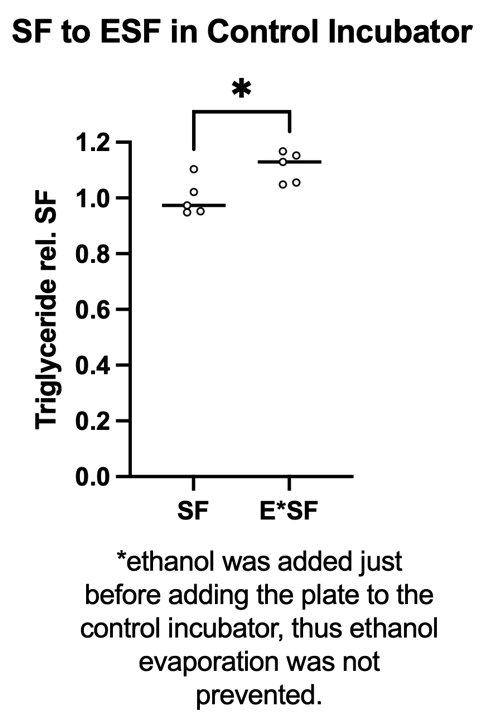 |
| --- | --- |
| **Supplementary Figure 2. Optimization of fatty acid overload experiments.** A) Individual spheroid triglyceride quantification relative to the control group after 6 days of treatment. Induction supplement was used in HepaRG media preparations to recapitulate metabolic-stress induced lipid accumulation at the concentration of oleic acid used throughout the study (300uM) and at a higher concentration (1000uM). One way ANOVA with Fisher’s LSD post-hoc analysis was used for analysis. B) Triglyceride content relative to the SF group. E* indicates that ethanol (50mM) was added to the SF media immediately prior to incubation, and the spheroids were placed in the same incubator as the SF group with no vaporized ethanol to maintain the ethanol concentration throughout the 6 days of treatment. Oleic acid concentration was 300uM in both groups. An unpaired t-test was used for analysis. *p<0.05, **p<0.01, ***p<0.001, and ****p<0.0001. | |

| 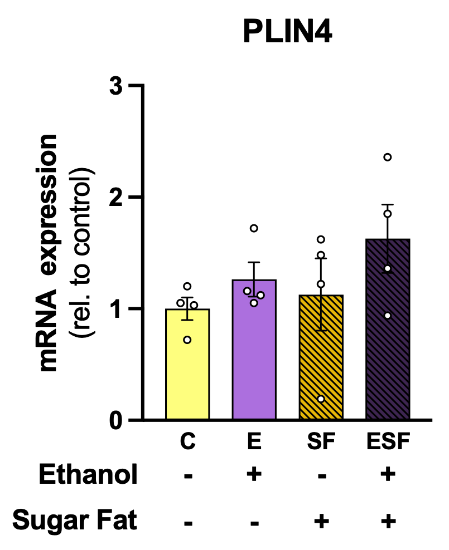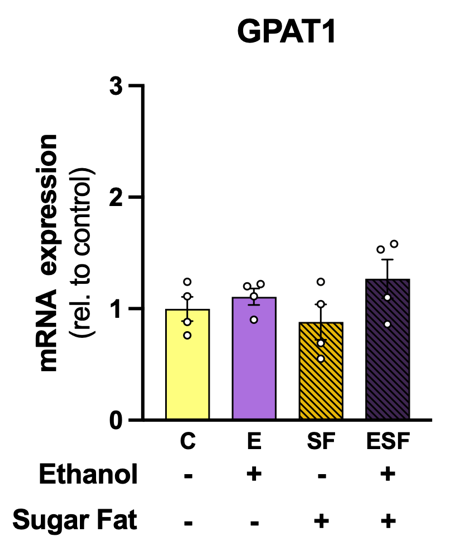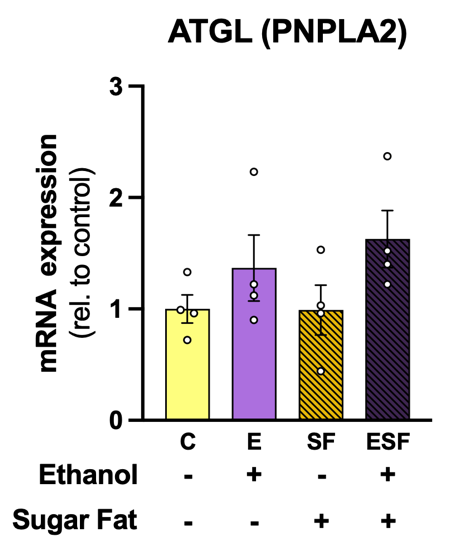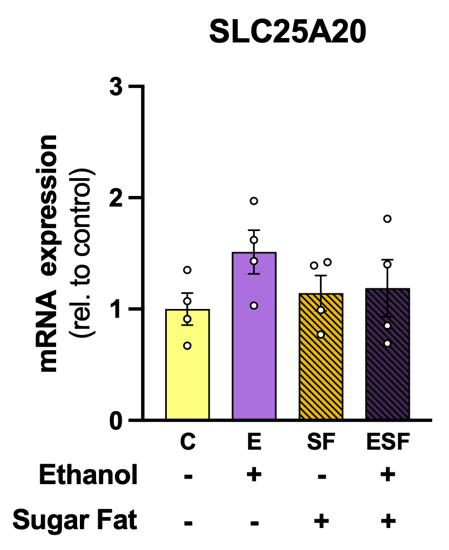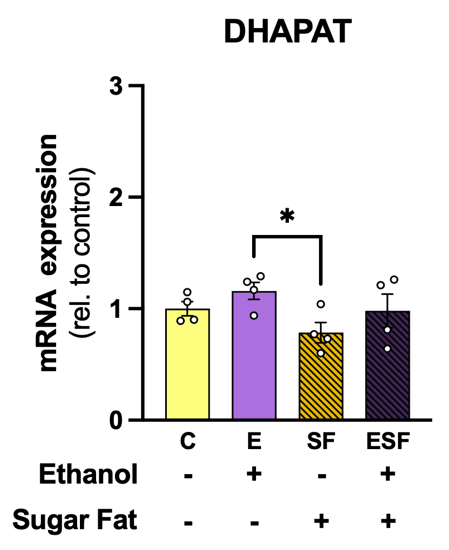 |
| --- |
| **Supplementary Figure 3. Genes involved in lipid homeostasis not significantly altered by ethanol or sugar-fat in HepaRG spheroids.** Each point represents 20-25 pooled spheroids from 4 independent experiments. Abbreviations: perilipin 4, PLIN4; glycerol-3-phosphate acyltransferase, mitochondrial, GPAT1; adipose triglyceride lipase (patatin-like phospholipase domain-containing protein 2), ATGL (PNPLA2); solute carrier family 25 member 20 (carnitine/acylcarnitine translocase), SLC25A20 (CACT); and dihydroxyacetone phosphate acyltransferase (glycerophosphate O-acyltransferase), DHAPAT (GNPAT). Two-way ANOVA with Fisher’s LSD post-hoc analysis was used for analysis. *p<0.05. |

| 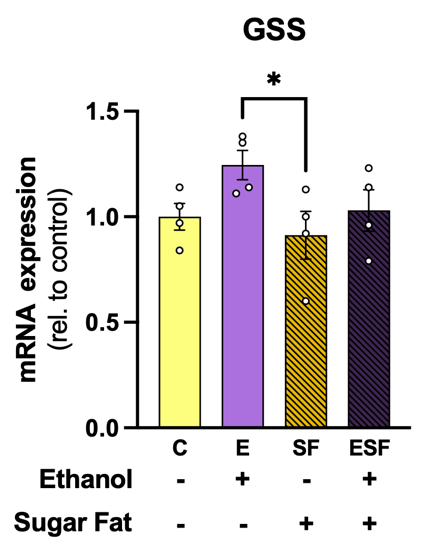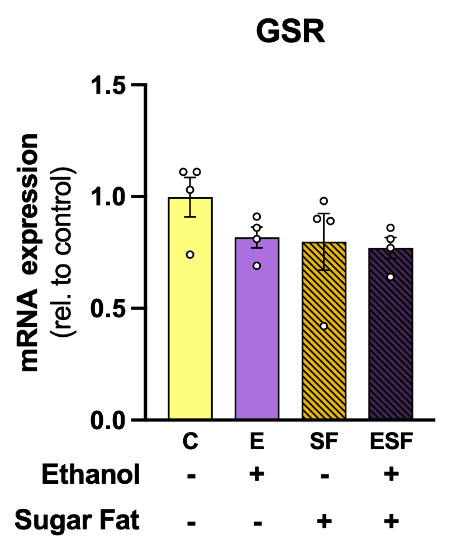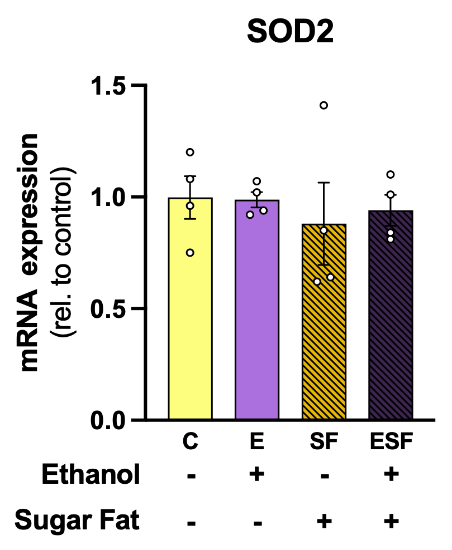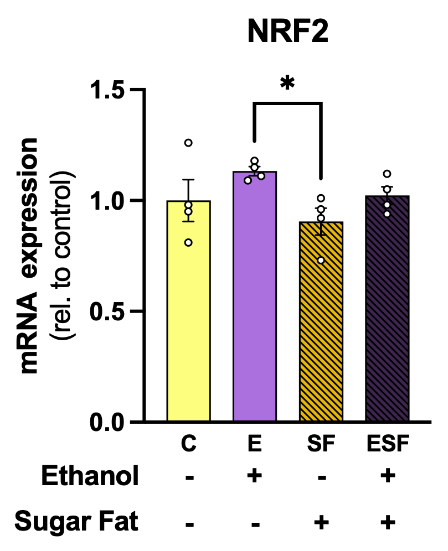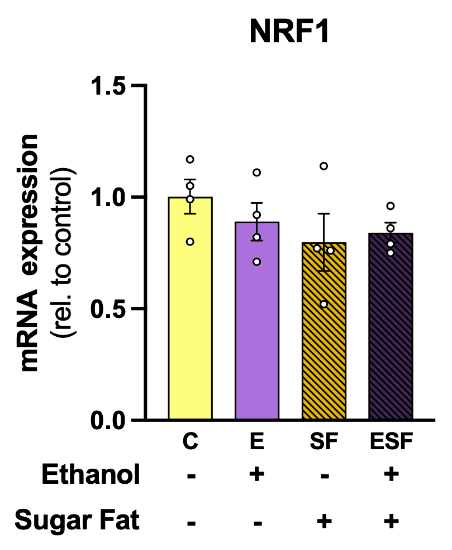 |
| --- |
| **Supplementary Figure 4. Genes involved in mitochondrial function and reactive oxygen species regulation that were significantly altered by ethanol or sugar-fat in HepaRG spheroids.** Each point represents 20-25 pooled spheroids from 4 independent experiments. Abbreviations: nuclear respiratory factor 1, NRF1; Nuclear factor erythroid 2-related factor 2, NRF2; superoxide dismutase 2, SOD2; glutathione-disulfide reductase, GSR; and glutathione synthetase, GSS. Two-way ANOVA with Fisher’s LSD post-hoc analysis was used for analysis. *p<0.05. |

| 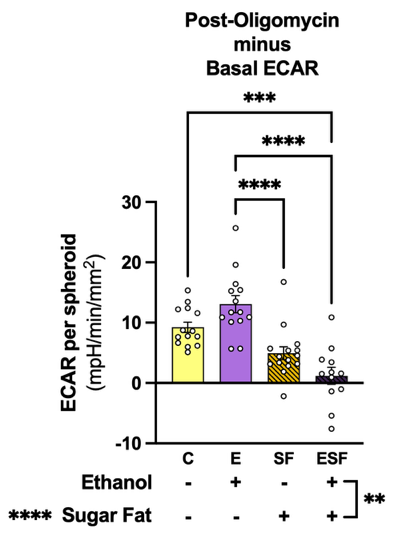 |
| --- |
| **Supplementary Figure 5. Ethanol and metabolic stressors interact to decrease the change in ECAR after ATP synthase is inhibited in the Mito Stress Test.** Each n represents ECAR corrected to mpH/min/spheroid and normalized to average spheroid area (mm^2^) across three independent experiments. Two-way ANOVA with Tukey’s post-hoc (n>5 per group) comparisons were used for analysis. *p<0.05, **p<0.01, ***p<0.001, and ****p<0.0001. |
